# Retraction Note: Circular RNA hsa_circ_0000517 modulates hepatocellular carcinoma advancement via the miR-326/SMAD6 axis

**DOI:** 10.1186/s12935-024-03474-3

**Published:** 2024-08-09

**Authors:** Shuwei He, Zhengwu Guo, Qian Kang, Xu Wang, Xingmin Han

**Affiliations:** 1 Department of Nuclear Medicine, The First Affiliated Hospital of Zhengzhou University, Zhengzhou, Henan 450000 China; 2 Henan Medical Key Laboratory of Molecular Imaging, No. 1 Jianshe East Road, Zhengzhou, Henan 450000 China

**Retraction Note: Cancer Cell International (2020) 20:360**

10.1186/s12935-020-01447-w

The authors have retracted this article. After publication, the authors found that the cell lines used in the experiments presented in this article were contaminated with HeLa cervical cancer and unidentified mouse cells, which was confirmed by STR profiling. This undermines the results and conclusions of this article.

All authors agree to this retraction.

